# Embryonic developmental process and clinical anatomy of the preperitoneal fascia and its clinical significance

**DOI:** 10.1007/s00276-022-03046-y

**Published:** 2022-11-20

**Authors:** Zheqi Zhou, Likun Yan, Yi Li, Jinsong Zhou, Yanbing Ma, Cong Tong

**Affiliations:** 1 Department of General Surgery, Shaanxi Provincial People’s Hospital, Xi’an, 710068 China; 2 Yan’an University, Yan’an, China; 3 Department of Human Anatomy, Histology and Embryology, School of Basic Medical Sciences, Xi’an Jiaotong University Health Science Center, Xi’an, 710061 Shaanxi China

**Keywords:** Inguinal hernia, Preperitoneal space, Intermediate mesoderm, Preperitoneal fascia, Urogenital fascia, Gubernaculum

## Abstract

**Purpose:**

Many researchers have different views on the origin and anatomy of the preperitoneal fascia. The purpose of this study is to review studies on the anatomy related to the preperitoneal fascia and to investigate the origin, structure, and clinical significance of the preperitoneal fascia in conjunction with previous anatomical findings of the genitourinary fascia, using the embryogenesis of the genitourinary system as a guide.

**Methods:**

Publications on the preperitoneal and genitourinary fascia are reviewed, with emphasis on the anatomy of the preperitoneal fascia and its relationship to the embryonic development of the genitourinary organs. We also describe previous anatomical studies of the genitourinary fascia in the inguinal region through the fixation of formalin-fixed cadavers.

**Results:**

Published literature on the origin, structure, and distribution of the preperitoneal fascia is sometimes inconsistent. However, studies on the urogenital fascia provide more than sufficient evidence that the formation of the preperitoneal fascia is closely related to the embryonic development of the urogenital fascia and its tegument. Combined with previous anatomical studies of the genitourinary fascia in the inguinal region of formalin-fixed cadavers showed that there is a complete fascial system. This fascial system moves from the retroperitoneum to the anterior peritoneum as the preperitoneal fascia.

**Conclusions:**

We can assume that the preperitoneal fascia (PPF) is continuous with the retroperitoneal renal fascia, ureter and its accessory vessels, lymphatic vessels, peritoneum of the bladder, internal spermatic fascia, and other peritoneal and pelvic urogenital organ surfaces, which means that the urogenital fascia (UGF) is a complete fascial system, which migrates into PPF in the preperitoneal space and the internal spermatic fascia in the inguinal canal.

## Introduction

Inguinal hernia is one of the common surgical diseases, and at least 20 million patients with inguinal hernia are treated with inguinal hernia repair worldwide every year [37]. The occurrence of an inguinal hernia is mainly related to the increase of abdominal pressure and the weakness of the abdominal wall. Since the beginning of human upright walking, the weakness of the abdominal wall structure in the inguinal region has laid the foundation for the occurrence of inguinal hernia [56]. Among the many ways to treat inguinal hernia, surgical repair of inguinal hernia is the most effective way to treat inguinal hernia, and there are many surgical approaches to inguinal hernia repair since Roman times [43], but whether open inguinal hernia repair or laparoscopic inguinal hernia repair, whether through the peritoneal approach (posterior approach) or the inguinal approach (anterior approach), the dissection of the abdominal wall in the inguinal region is crucial. After the discovery of Bogros' space between the iliac fascia, the transversalis fascia, and the mural peritoneum by Bogros in 1823, Retzius discovered Retzius' space in 1858, which is located in front and on both sides of the bladder [47]. As a result, the preperitoneal space, which is rich in adipose tissue and has a complex fascial structure, has become a hot topic of research in basic membrane anatomy. Within the preperitoneal space, the complex fascial structure between the wall peritoneum and the transversalis fascia, namely the preperitoneal fascia (PPF), is an important and confusing fascial structure that has not been uniformly and holistically recognized. The author believes that the main reason for the confusion about this structure is the lack of research on its embryonic origin and embryonic dynamic developmental processes. To better understand the origin, structure, and relationship of the preperitoneal fascia to the genitourinary fascia and to explore its clinical significance, the origin of the preperitoneal fascia is described through a review of the literature, guided by the embryonic development of the urogenital system and its fascial structures, in conjunction with previous anatomical studies. The idea that the preperitoneal fascia is part of the urogenital fascia in the preperitoneal space and extends into the inguinal canal to become the internal spermatic fascia is proposed. Ultimately, the anatomical relationship between the urogenital fascia (UGF) and the anterior peritoneal fascia is presented for consideration and application in the transabdominal preperitoneal (TAPP) procedure.

## Methods

### Analysis of publications

Studies concerning the structure, origin, nomenclature, stratification, embryonic development of the preperitoneal fascia, and the anatomical structure of the posterior wall of the inguinal canal in the inguinal region are reviewed, with emphasis on studies concerning the anatomical structure of the preperitoneal fascia and its association with the embryonic development of the retroperitoneum. Through literature review, the origin of the preperitoneal fascia was explained in the context of previous anatomical studies, using the embryonic development of the urogenital system and its fascial structures as a guide, and the idea that the preperitoneal fascia is part of the urogenital fascia (UGF) in the preperitoneal space and extends into the inguinal canal to become the internal spermatic fascia was proposed. The final thoughts and applications of the TAPP procedure are presented.

### The study of dissection

The study was based on the dissection of ten formalin-fixed cadavers (9 males and 1 female) provided by the Department of Anthropotomy and Histo-Embryology of Xi'an Jiaotong University Health Science Center, using one set of anatomic devices and one camera. Written informed consent was obtained from the immediate family members of the deceased for educational and scientific research purposes. The format of the informed consent form is in line with the guidelines of the China Organ Donation Administrative Center. We performed a fine dissection of retroperitoneal and extraperitoneal structures in the inguinal region on the ten formalin-fixed cadavers mentioned above. Each cadaver was incised horizontally from the anterior superior iliac spine to the medial umbilical fold, and the deep peritoneal fascial tissues were carefully separated, and the vas deferens and its spermatic vessels were carefully dissected to the root of the sigmoid mesentery in male cadavers to fully expose the inguinal hernia repair area. The peritoneum was incised longitudinally at the bilateral paracolic sulcus, the colon was lifted to expose the retroperitoneal space, and the retroperitoneal renal fascia and presacral fascia were carefully dissected. The continuation of the retroperitoneal fascia, pelvic fascia, and anterior peritoneal fascia was observed.

## Results

### Anatomical study findings

**Fig. 1:**
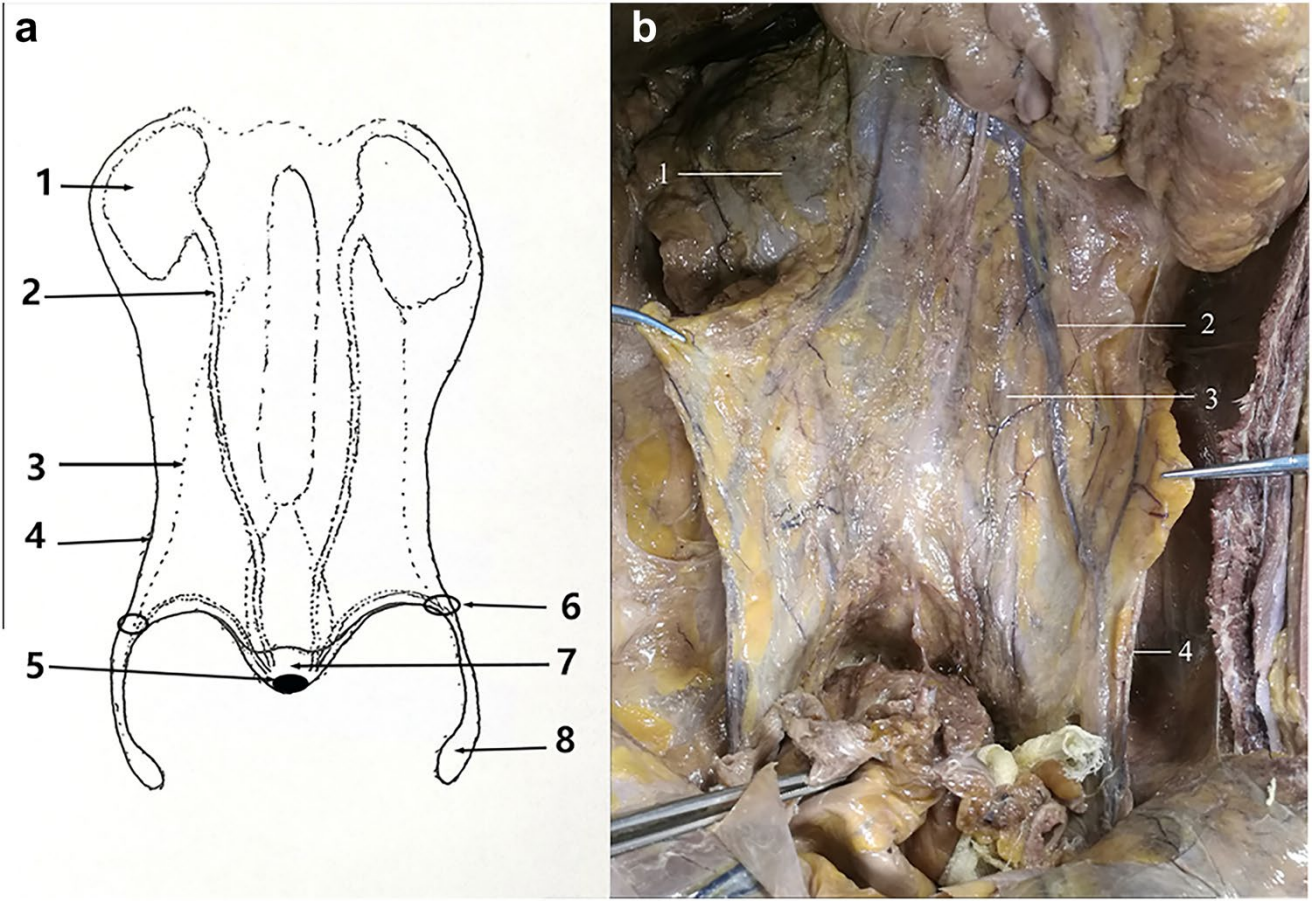
The overall appearance of the UGF. **a** Schematic diagram of the anatomical composition of the UGF. The solid line is the exact boundary of the UGF; The dashed line is the uncertain boundary of the UGF. **b** The partial entity of panel a in a cadaver. 1, kidney; 2, ureter; 3, genital vessels; 4, the lateral boundary of the UGF; 5, prostate; 6, deep inguinal ring; 7, urinary bladder; 8, spermatic sheath

**Fig. 2:**
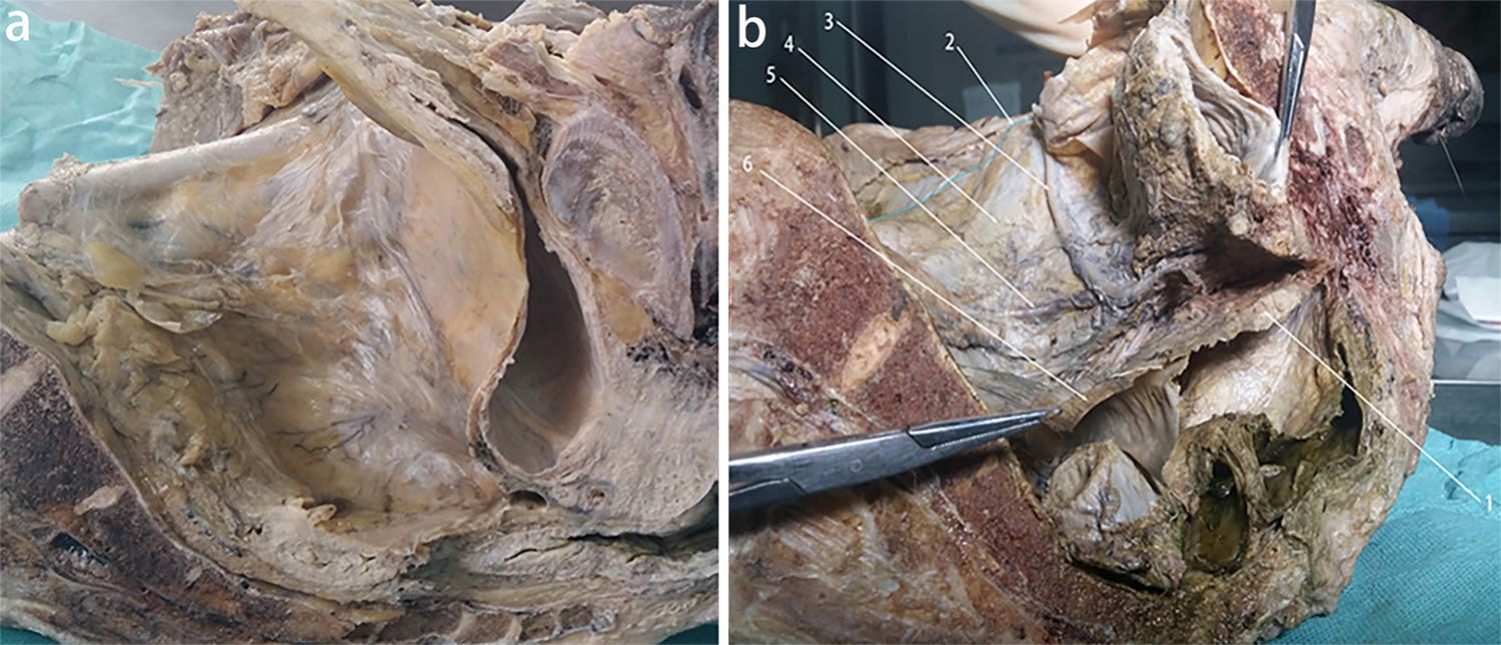
Coronal anatomy of the urogenital fascia and the preperitoneal fascial migration. **a** Pelvic anatomy without removal of the peritoneum. **b** Anatomy showing migration of the urogenital fascia into the anterior peritoneal space after removal of the peritoneum. 1 Denonvilliers fascia, 2 lateral border of the genitourinary fascia, 3 vas deferens, 4 the displaced portion of the urogenital fascia in the preperitoneal space, the preperitoneal fascia (PPF), 5 ureters, 6 peritoneum

We performed a dissection guided by the embryonic developmental process of the intermediate mesoderm and the urogenital system. A complete fascial system was found to exist in the retroperitoneal, pelvic, and preperitoneal spaces (Fig. 1). This fascial system surrounds the kidney, ureter, vas deferens, spermatic cord blood vessels, hypogastric nerves, seminal vesicle gland, prostate, and bladder. The migrating part of this fascia system in the preperitoneal space is the PPF, and we refer to this fascial system as the urogenital fascia (UGF) (Fig. 2), which is more consistent with the holistic view of embryonic development and what is seen intraoperatively.

### Description of publications

Traditional anatomy considers the anatomical structure of the posterior wall of the inguinal canal to consist of the transversalis fascia, extraperitoneal tissue, and parietal peritoneum. With the development of basic anatomy and minimally invasive surgical techniques, it was gradually discovered that the fascial structures within the preperitoneal space were complex. In 1844, Cooper illustrated the PPF behind the transversalis fascia, and in 1975, Fowler [28] further clarified the structure of the PPF and illustrated its location. Later, Mirilas [48] further proposed that the PPF divides the preperitoneal space into the parietal plane between the transversalis fascia and the PPF and the visceral plane between the PPF and the peritoneum. In 1977, Arregui [5] found through laparoscopic dissection of the inguinal region that the blood supply of the so-called double-layered membranous structures of the transversalis fascia does not have the same source, and the first structure seen by laparoscopic incision of the peritoneum should be the PPF rather than the transversalis fascia, and that the PPF is an independent anatomical level [4], a view shared by some scholars [54, 58]. There are also various controversies regarding the origin, structure, nomenclature, and starting and ending points of the PPF [6–8, 13, 39, 45]. Japanese scholar N. Asakage believed that the structural origin of this fascia belongs to a part of the retroperitoneal fascia [9], and other scholars believed that within the preperitoneal space, the transversalis fascia, PPF, umbilical prevesical fascia, and anterior bladder fascia are the same structure [36, 49].

The first 8 weeks of embryonic development are the early stages of human embryonic development. During this period, the fertilized ovum undergoes various stages of development from mulberry embryo to blastocyst through proliferation and differentiation in sequence. As the ectoderm grows faster, the edge of the embryonic disc gradually curls ventrally to form the embryonic body, and the intra-embryonic body cavity is formed. The mesoderm around the notochord can be divided from the inside out into the paraxial mesoderm, the intermediate mesoderm, and the lateral mesoderm [26]. During the embryonic development of the urogenital system, the development of the intermediate mesoderm and the cloaca is crucial, with the intermediate mesoderm developing as part of the organs of the urogenital system and their membrane [22, 42] and the cloaca serving as the primordium of the bladder and prostate [17]. The mesonephric duct formed by the intermediate mesoderm at the beginning of the 4th week of embryonic development extends caudally and opens into the cloaca at the caudal end of the primitive gut [59]. In the 5th week of human embryonic development, the cloacal side of the mesonephric duct sends ureteral buds to the dorsal side of the embryo and extends into the caudal end of the mesonephric ridge [42, 46], inducing differentiation into the metanephrogenic tissue, i.e., the human permanent kidney. The above embryonic developmental process verifies that the renal fascia accompanies the development of the urogenital system to migrate to the pelvis, which is similar to the idea proposed by Muntean [50], Diarra [21] and Yi Li [41] through dissection. Yang [61] et al. observed the continuation of the renal fascia into the pelvis and wrapped the ureter, reproductive vessels, and hypogastric nerves, calling it the urogenital–hypogastric sheath. Duhamel [23] and N. Asakage [6] suggested that the fascial system derived from the renal fascia covers the bladder and migrates into the preperitoneal fascia and the medial umbilical fold in the preperitoneal space. Sato [54] similarly suggested that the PPF is continuous with the renal fascia.

During embryonic development, the anatomical position of the gonad changes in close correlation with hormone levels [34]. The gonads that develop from the intermediate mesoderm gradually grow and are suspended from the surface of the genital ridge by the cranial suspensory ligament (also called the cranial mesonephric ligament or the diaphragmatic ligament) [60]. During the 7th and 8th weeks of male embryonic development, the cranial suspensory ligament between the genital ridge and the testis gradually disappears [20, 40, 51]. In its place, the caudal end of the gonad gradually forms a small nodule known as the inguinal ligament of the gonad, which is near the testis and epididymis and is called the gubernaculum. During the 7th week of gestation [10, 32], the testes and epididymis migrate toward the inguinal region under the pull of the gubernaculum and eventually cross the inguinal canal into the scrotum during the 25^th^–35th weeks of gestation. During the migration of the testes to the scrotum, the structural complexity of the tubercle, testes, and epididymis, which is derived from the intermediate mesoderm, pushes the peritoneum into the inguinal canal to form the vaginal process. At the same time, the extraperitoneal muscles and their fascial structures wrap around this structural complex to form the cremaster muscle and its aponeuroses [33]. In contrast, in the female fetus, the cranial suspensory ligament transforms into the suspensory ligament of the ovary [10], which pulls the ovary and eventually stays in the pelvis, and the round ligament of the uterus enters the inguinal canal [12]. One researcher [35] observed by cross-sectional specimens of paraffin-embedded retroperitoneal tissues of human embryos after 29 HE staining procedures that there was an opposing fascia parallel to the renal fascia. This opposing fascia and the renal fascia contain a large amount of adipose tissue that surrounds the retroperitoneal organ and the organ itself. Matsubara [44] et al. also found a fascial structure extending toward the inguinal region after leaving the renal fascia by light microscopic observation of a specimen of retroperitoneal tissue of a fetus at 20 weeks of gestation. Hayes [30] suggested that there are three types of development of retroperitoneal fasciae: migration fasciae, fusion fasciae, and parietal fasciae, with migration fasciae, arising from the migration and growth of the organ during embryonic development, which gradually exerts pressure on the loose connective tissue surrounding the organ, causing the fibers in the loose connective tissue to linearly orient and eventually compress into a fascia-like structure.

Some authors [62] studied the structure of the spermatic cord by microanatomy and 3D reconstruction techniques and found that the spermatic cord contains two sets of fascial systems. These two fascial systems encircle the internal vascular plexus of the spermatic cord and the vas deferens and its accessory vessels, respectively. The fascial system that surrounds the vascular plexus within the spermatic cord represents the traditional anatomy of the internal spermatic fascia, while the fascia encircling the vas deferens and its accessory vessels has no name. Stoppa et al. [57] demonstrated that the UGF extends in a lateral direction and wraps around the vas deferens and the genital vessels, called the spermatic sheath, in the transperitoneal anterior repair of the male inguinal hernia. The spermatic sheath reaches the deep inguinal ring laterally and continues laterally to the anterior abdominal wall. In females, they observed that the UGF extends laterally around the round ligament of the uterus and the ovarian vessels and extends downward laterally, and the lateral extension of the UGF is referred to as the genital sheath. The findings of Folscher [27] et al. are very similar to those of Stoppa. Lipomas in the inguinal canal usually include the spermatic cord lipoma and the round ligament lipoma, and lipomas in the inguinal canal are not true tumors, but a continuation of extraperitoneal fat [25]. If one assumes that the internal spermatic fascia is the continuation of the UGF in the inguinal canal, then the spermatic cord lipoma must be continuous with the fat within the UGF. Heller et al. [31] found that the spermatic cord lipoma in the inguinal canal was connected to the PPF through the deep inguinal ring of the inguinal canal by dissection, and concluded that the spermatic cord lipoma was formed by the PPF entering the inguinal canal under the action of gravity and was not a true lipoma. The nourishing vessels of the spermatic lipoma are derived from PPF and reside in the same adipose tissue structure as the accessory vessels, lymphatic vessels, and nerves around the spermatic cord, and it is difficult to separate the spermatic cord lipoma from its intraoperatively, and blunt separation is often used to protect the accessory structures around the spermatic cord [38].

## Discussion

The anatomical studies related to the PPF have not been uniformly understood [4–8, 13, 36, 39, 45, 49, 56, 58], with some scholars suggesting that the PPF is derived from the transversalis fascia and others suggesting that the PPF is an independent fascial structure. Most of the relevant studies have been confined to the anterior peritoneal space, which limits the understanding of the structure of the anterior peritoneal fascia. The Japanese scholar N. Asakage [9] has suggested that the anterior peritoneal fascia originates from the retroperitoneal fascia through the relationship of fascial embryonic development and that the human fascial system gradually extends from the embryonic development and undergoes a migration in position, and is a whole with the same origin. Understanding the embryonic developmental process of the intermediate mesoderm and urogenital organs can clearly recognize the connection between the retroperitoneal fascia and PPF in the anterior peritoneal space. This review will discuss the relationship between the retroperitoneal fascia, the internal spermatic fascia, and the anterior peritoneal fascia in the context of the embryonic developmental processes of the intermediate mesoderm and urogenital organs and anatomical findings. It will also discuss the application of UGF in the TAPP procedure.

### Association of the preperitoneal fascia with the intermediate mesoderm-cloaca developmental process

**Fig. 3:**
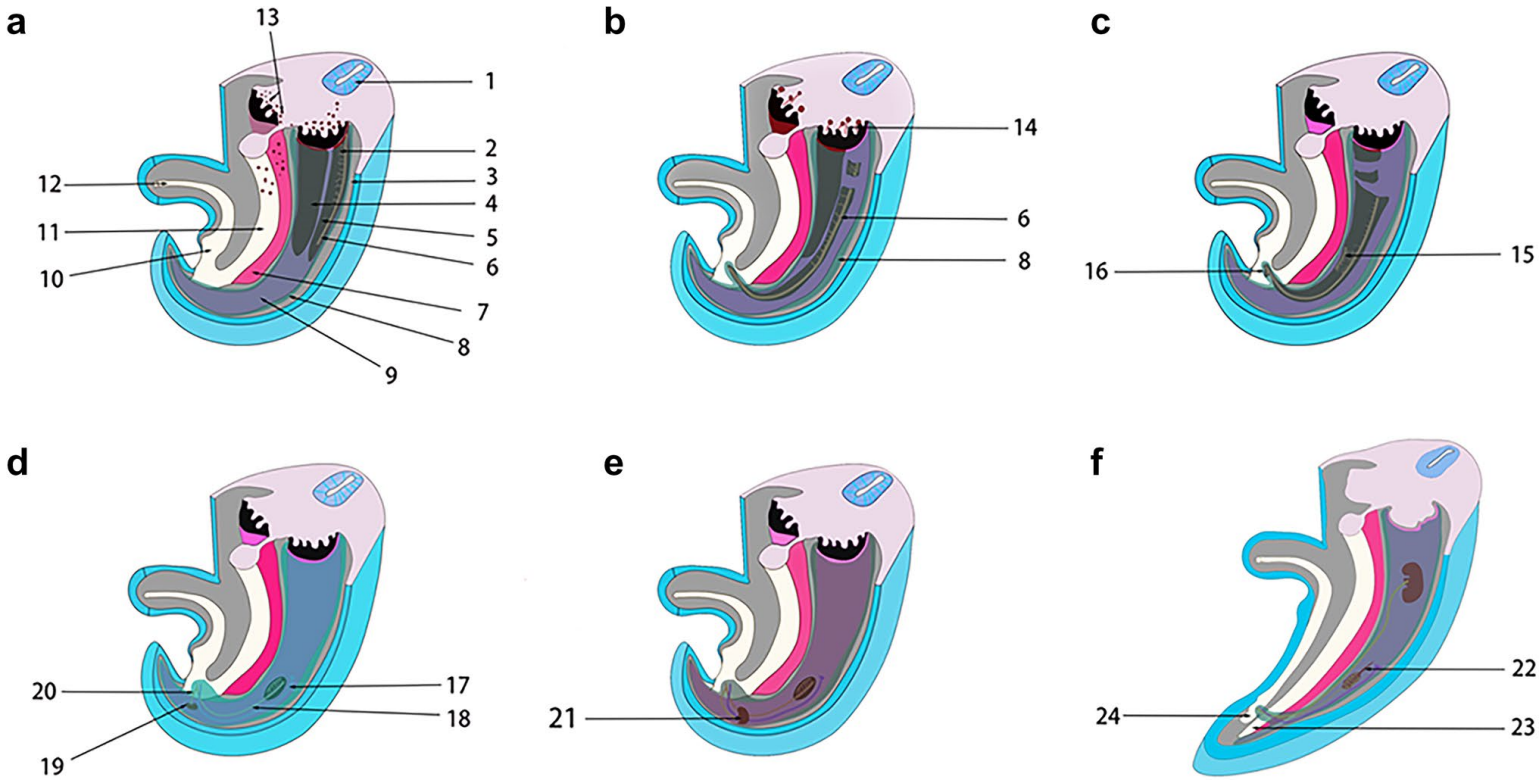
The UGF with dynamic development and migration of the genitourinary system. This figures shows the dynamic migration of UGF from the retroperitoneum to the cloaca, accompanied by and wrapped around the urogenital organs derived from the intermediate mesoderm during human embryonic development. **a** The gradual development of the gonadal and mesonephric ridge in the intermediate mesoderm at the beginning of the 4th week of human embryonic development as the primordium of the urogenital organs. **b** The intermediate mesoderm of UGF from the mesonephric duct to the cloaca at the end of the 4th week of human embryonic development. **c**, **d** During the 5th and 6th weeks of human embryonic development, the UGF wraps around the ureteric bud and gradually develops into the ureter, and migrates back into the intermediate mesoderm to develop into the kidney. **e**, **f** During the 7th week of human embryonic development, the kidney gradually moves up and the gonad gradually moves down. 1 Neural tube, 2 mesonephric tubule, 3 parietal mesoderm, 4 genital ridge, 5 mesonephric ridge, 6 mesonephric duct, 7 dorsal mesentery of hindgut digestive tract, 8 UGF, 9 intermediate mesoderm, 10 cloaca, 11 hindgut, 12 allantois, 13 Primordial germ cells, 14 primary sex cord, 15 secondary sex cord, 16 ureteric bud, 17 paramesonephric duct, 18 vas deferens, 19 metanephrogenic tissue, 20 ureter, 21 primitive kidney, 22 cranial suspensory ligament (also called the cranial mesonephric ligament or the diaphragmatic ligament), 23 primitive rectum, 24 primitive bladder

**Fig. 4:**
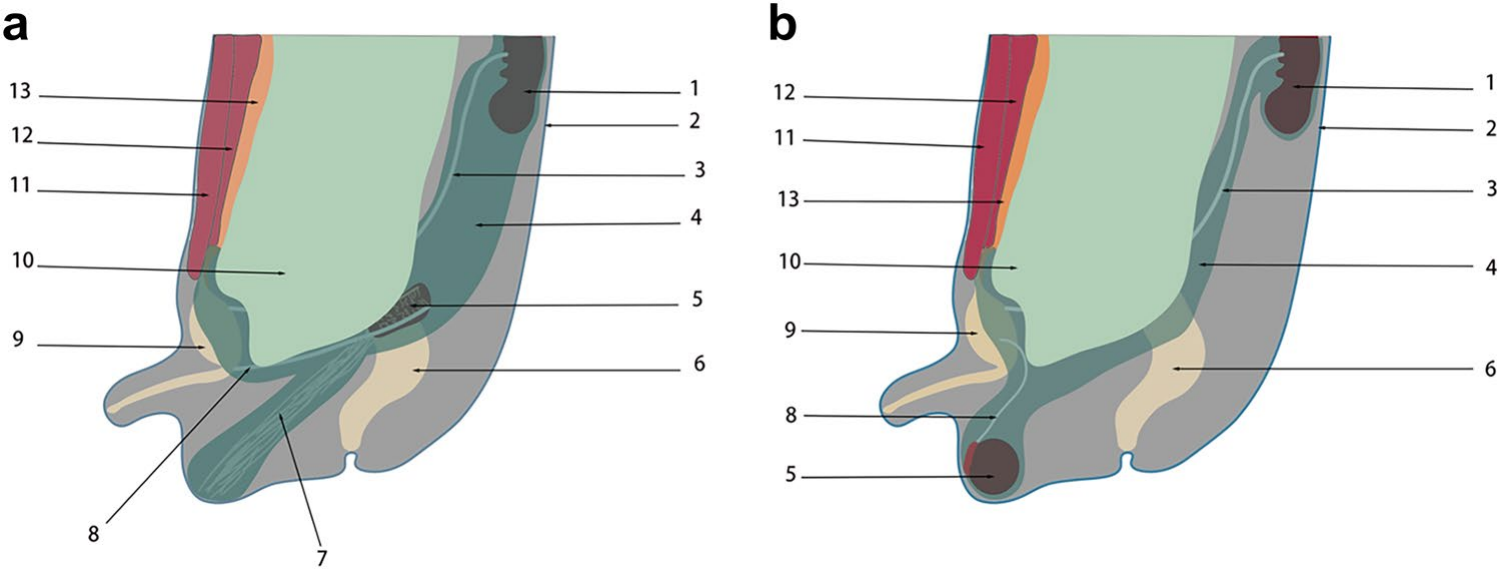
Distribution diagram of the UGF in retroperitoneal, pelvic, and preperitoneal spaces. This figure shows that UGF accompanies the embryonic development of the urogenital organs from the retroperitoneum to the scrotum, the bladder, and the preperitoneal space. 1 Kidney, 2 skin and superficial fascia, 3 ureter, 4 UGF, 5 testis, 6 rectum, 7 gubernaculum, 8 vas deferens, 9 bladder, 10 peritoneum, 11 obliquus internus abdominis, 12 rectus abdominis muscle, 13 transversalis fascia

The embryonic development process of intra-abdominopelvic urogenital organs derived from the intermediate mesoderm can be understood as the migration of the mesonephric ducts within the intermediate mesoderm to the cloaca, and the return migration of the ureters from the cloaca to the intermediate mesoderm in two stages (Fig. 3). Between the retroperitoneal mesoderm and the cloaca, the fascia system derived from the intermediate mesoderm accompanies the migration of the mesonephric duct and ureter from the retroperitoneum through the pelvis to the preperitoneal space (Fig. 4) [17, 22, 26, 42, 46, 59]. Researches by some scholars are consistent with the author's view that the development of the entire urogenital system reveals a dynamic migration process of tissue originating from the intermediate mesoderm to the pelvic and inguinal regions [6, 21, 23, 50, 54, 61]. The fascial structures originating from the intermediate mesoderm accompanied and encircled the ureter and vas deferens and their vessels, nerves, and lymphatics, and gradually migrated between the peritoneum and the transversalis fascia to form a loose fascial tissue, that is PPF, under the migration of the mesonephric ducts and ureter and the traction of the testis and introitus structures developed from the mesoderm, as well as the twisting, folding, fusion, and extrusion of the gubernaculum structures during the embryonic development.

### Understanding the origin of the preperitoneal fascia from the embryonic development of the gonads

**Fig. 5:**
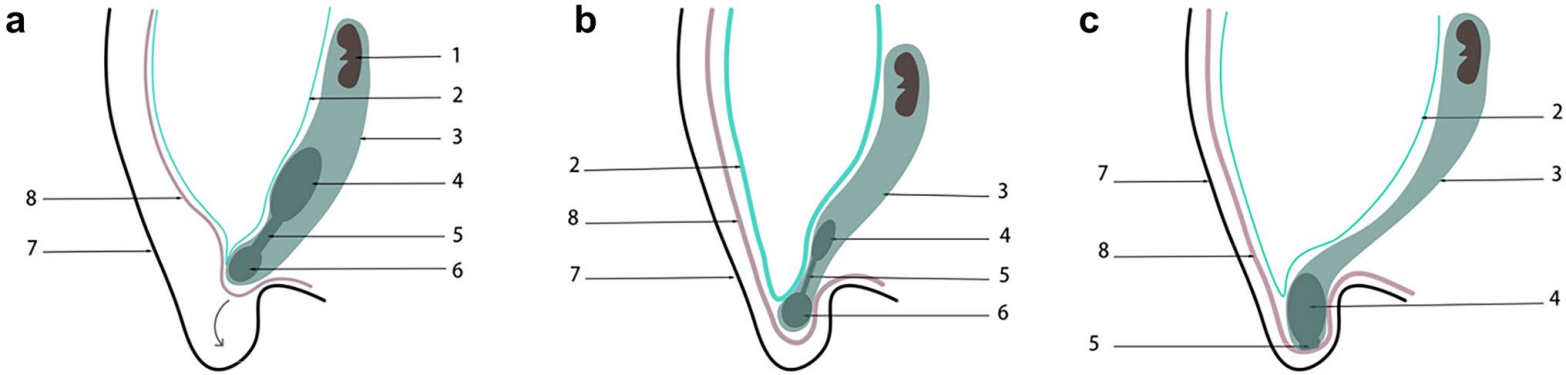
Diagram of the testicular descent process. 1 kidney, 2 peritoneum, 3 UGF, 4 testis, 5 gubernaculum, 6 tubercle, 7 skin, 8 transversalis fascia

The migration of the male fetal testis and epididymis from the gonad in the retroperitoneal to the anterior peritoneal space and the development of the round ligament of the female fetus have implications for understanding the distribution, structure, and origin of UGF [10, 12, 20, 32, 33, 40, 51, 60]. By understanding the migration process of the gonads and the anatomy of the retroperitoneal fascial system, we can conjecture that the lax adipose tissue of the retroperitoneum wrapping the kidney and ureter completes the migration process from the retroperitoneal perinephric area to the preperitoneal space along with the testis (Fig. 5). By dissecting the retroperitoneal fascia of human embryos, some scholars found similar [35, 44]. The migration fasciae described by Hayes correspond to the process of occurrence and development of UGF [30], and it is evident that UGF belongs to migration fasciae. During human embryonic development, the UGF migrates from the retroperitoneum to the inguinal region along with the development of the testes, which is consistent with the intraoperative and dissection findings that the UGF contains the spermatic cord and its vascular nerves. Based on the understanding of the embryonic development of the gonads, the author believes that this structural complex, which originates from the intermediate mesoderm of the retroperitoneum, causes the UGF to wrap around the testis and its accessory vessels, nerves, and lymphatic vessels during the migration from the retroperitoneum to the inguinal region, resulting in the formation of the so-called PPF. In other words, the PPF is the localized structure of the UGF as a whole fascial system in the preperitoneal space. The embryonic development of the gonads described above supports the view that the migrating part of the UGF between the peritoneum and the transversalis fascia in the inguinal region is the PPF.

### Understanding the migration of the urogenital fascia into the inguinal canal from the structure of the spermatic cord

**Fig. 6:**
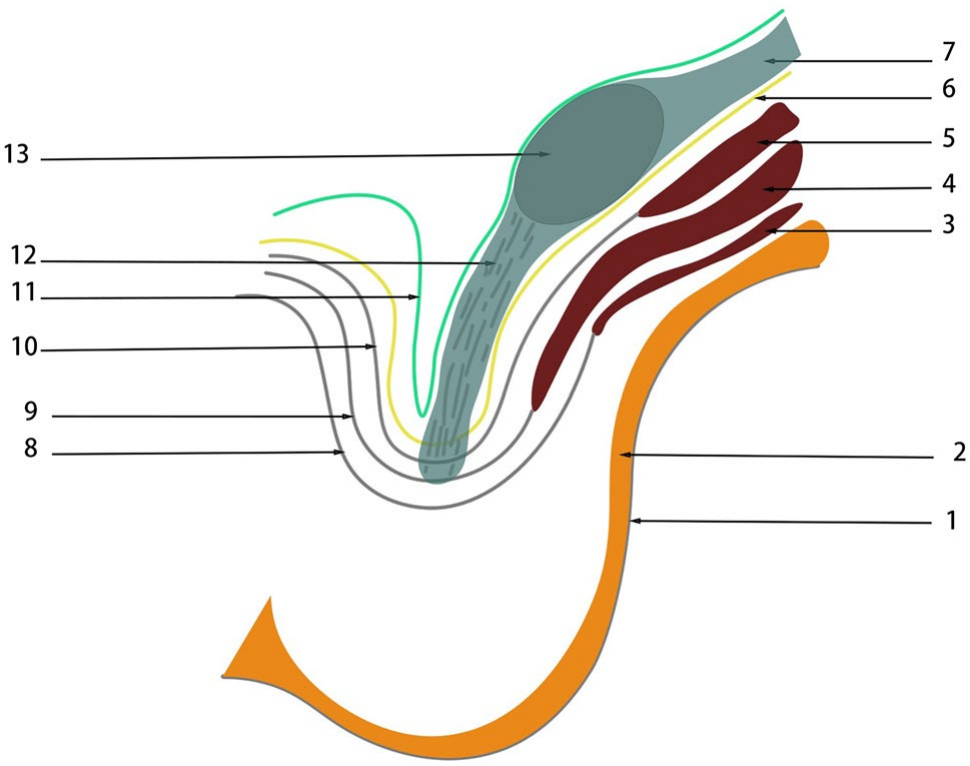
Coronal plan view of the scrotum. 1 scrotal skin, 2 superficial fascia, 3 external oblique abdominal muscle, 4 internal oblique abdominal muscle, 5 transverse muscle, 6 transversalis fascia, 7 UGF, 8 external oblique abdominal tendon membrane, 9 internal oblique abdominal tendon membrane, 10 transversalis tendon membrane, 11 peritoneum, 12 gubernaculum, 13 testis

Yang Yu et al. [62]. found the existence of a separate fascial system surrounding the vascular plexus of the spermatic cord and considered it to be the internal spermatic fascia, and named the fascia surrounding the vas deferens and its accessory vessels as the vas deferens fascia, which is inconsistent with the traditional anatomical definition of the internal spermatic fascia. The author believes the vas deferens fascia and the internal spermatic fascia are actually the same fascial structure, formed by the extrusion of the UGF when it enters the inguinal canal. That is, the UGF moves like a crumpled envelope inside the inguinal canal into the internal spermatic fascia, which wraps around the vas deferens and its blood vessels and spermatic vessels. Folscher [27] and Stoppa [56] agreed that UGF wraps around the vas deferens and reproductive vessels migrating toward the internal ring and continuing to the anterior abdominal wall. Based on the above findings we can assume that the internal spermatic fascia is a continuation of the UGF in the inguinal canal and that in the retroperitoneum the UGF forms the spermatic sheath in males. Within the inguinal canal migrates the internal spermatic fascia (Fig. 6). Heller et al. [31]. found by dissection that the spermatic cord lipoma is continuous with the PPF through the internal ring. It can be seen that intra-seminomatous adipose tissue, like PPF, is an adipose tissue located within the UGF, similar to the perirenal fat, and homologous to the perirenal fat, the fat around the bladder, and the fat within the medial umbilical fold. This strongly supports the assumption that UGF accompanies the testis into the scrotum in the preperitoneal space and migrates into the PPF and the internal spermatic fascia.

### Re-conceptualization of the anterior preperitoneal space and its internal fascia structure guided by a holistic view of embryonic development

**Fig. 7:**
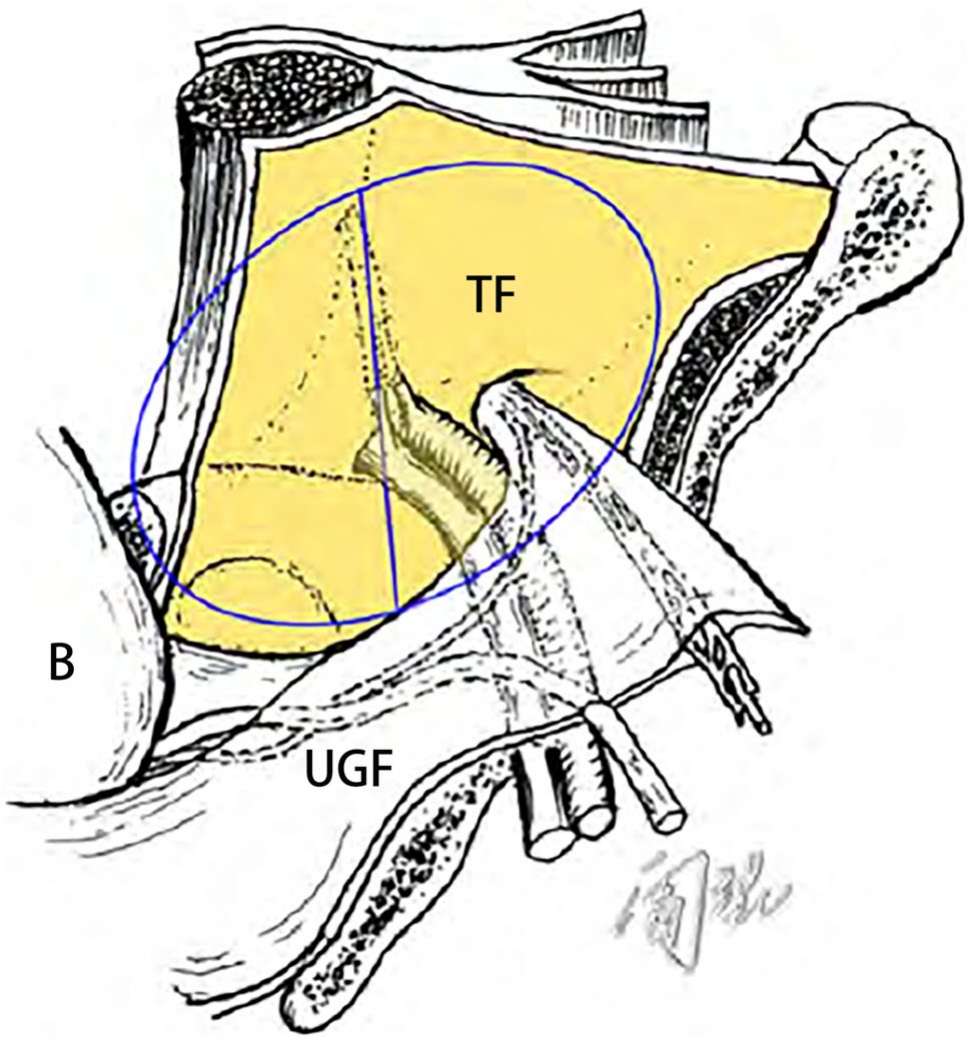
Distribution of the UGF in the preperitoneal space. *UGF* urogenital fascia, *TF* transversalis fascia, *B* bladder

**Fig. 8:**
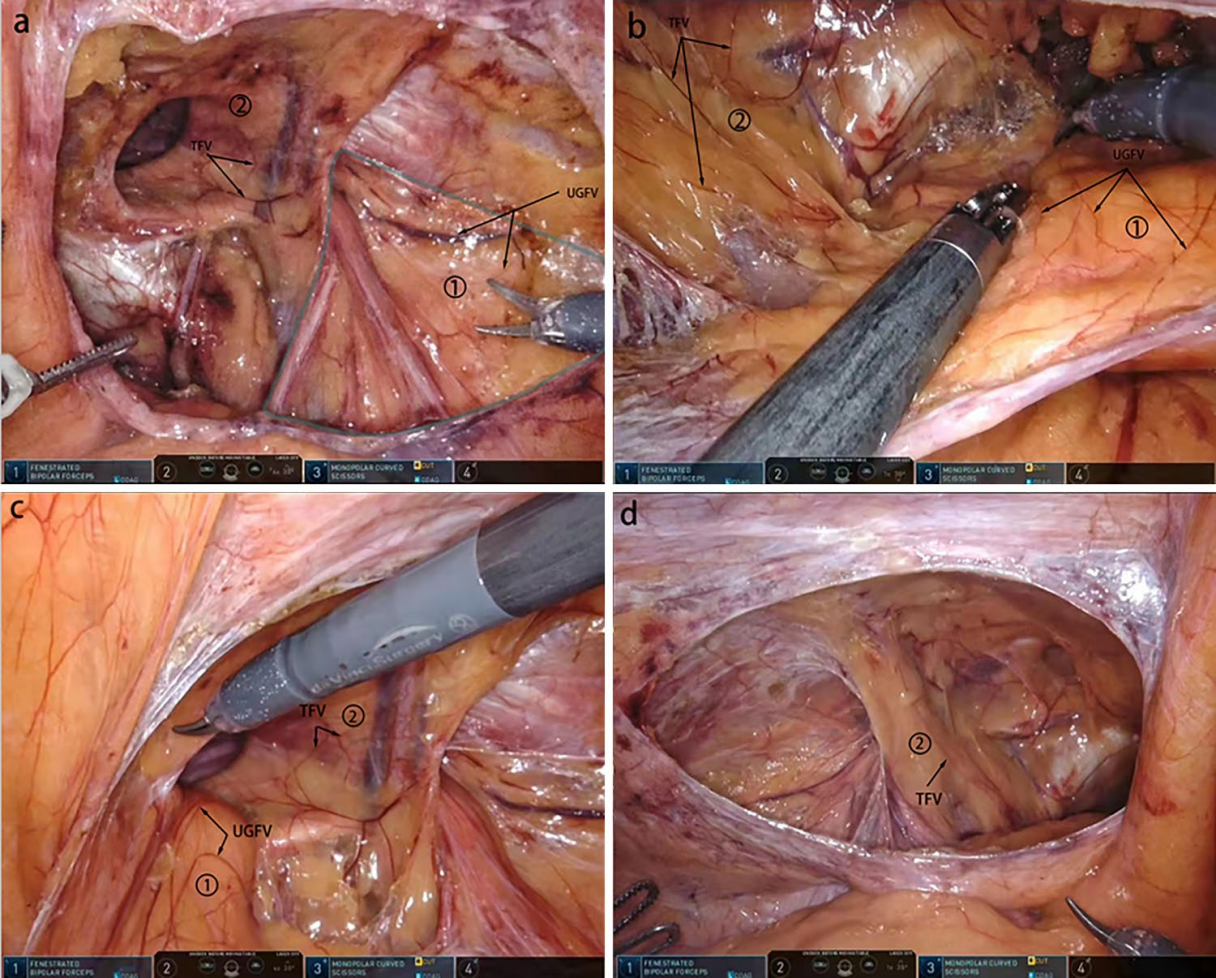
Distribution of blood vessels and adipose tissue in the UGF and the transversal fascia. The figure shows a set of photographs of the preperitoneal space taken during the laparoscopic transabdominal preperitoneal inguinal hernia repair (TAPP) procedure using the da Vinci robot. It can be seen that the vascularity of the transversalis fascia and the UGF has different orientations and different fat distribution. 1 urogenital fascia (UGF), 2 transversalis fascia (TF). **a** The distribution of the UGF in the preperitoneal space with a clear border. UGFV: vascularity of the urogenital fascia, TFV: vascularity of the transversalis fascia

During embryonic development, the migration of the mesonephric ducts from the intermediate mesoderm into the allantois (the original base of the bladder) and the return of the ureter to the intermediate mesoderm are two processes that allow the UGF to wrap around and accompany the development of the urogenital organs to complete the transfer from the retroperitoneum to the pelvis and finally terminate in the preperitoneal space. N. Asakage suggested that the preperitoneal space, as the gap between the abdominal wall muscles and the peritoneum, is formation is associated with the umbilical funiculus being brought into the abdominal wall during the 5th–6th weeks of embryonic development [9]. The author suggests that the bladder, as an extraperitoneal organ, develops between the peritoneum and the transversalis fascia in the lower part of the anterior abdomen and pushes out the parietal mesoderm to form the preperitoneal space, and the UGF then migrates into the space in a membranous form and migrates into the preperitoneal fascia. Based on the anatomy of UGF and embryonic development, we may assume that the ventral wall side of Bogros’ gap and Retzius' gap is composed of the transversalis fascia, while the peritoneal side is composed of bladder, UGF, and peritoneum. The UGF wraps around the vas deferens during embryonic development and enters the internal canal dividing the preperitoneal space into the Retzius’ space on the inside and the Bogros’ space on the outside, in other words, the Retzius’ space and the Bogros’ space are themselves the same gap that is the preperitoneal space (Fig. 7). The UGF then extends membranously between the bladder, the medial umbilical fold and the deep inguinal ring. Intraoperatively, the vascular orientation and tortuosity of the transversalis fascia and the UGF surface can be clearly observed to be different (Fig. 8). The vessels on the surface of the transversalis fascia were relatively straight, whereas those on the surface of the UGF were relatively tortuous, forming an interlaced vascular network. It is also observed intraoperatively that the fat distribution outside the transversalis fascia and within the UGF is different and not constant. Arregui [5] made detailed laparoscopic observations of the anatomical levels of the inguinal region and found that the blood supply to the superficial and deep layers of the transverse fascia is different under the “double layer doctrine”. This view is similar to that found in high-throughput gene microarray screening experiments, where several genes related to angiogenesis are substantially upregulated during the differentiation of embryonic stem cells to adipocytes, and in early morphological observations, where adipocytes were also found to be generated from connective tissue rich in capillary network, suggesting that embryonic adipogenesis is closely related to vascularity [14]. It is evident that the PPF, as part of the UGF in the preperitoneal space, has its different hematologic sources of fat from the fat in the transversalis fascia. However, the exact mechanisms remain to be investigated, and understanding these differences will help to better find and determine the correct anatomical plane.

### Experiencing the preperitoneal fascial structures in surgical practice

Adequate knowledge of the fascial structures in the inguinal region and deliberate intraoperative protection can help to reduce the volume of the hernia sac, define the plane of surgical manipulation, and reduce vascular and nerve injury [15]. The selection of the anatomical plane between the peritoneum and the UGF helps to protect the vessels and nerves during the laparoscopic inguinal hernia repair (LIHR) [19]. There is high variability in the distribution and arrangement of nerves in the inguinal region [52, 53]. The incidence of chronic pain after inguinal hernia repair (CPIP) is often increased if there is an inadvertent intraoperative injury, which seriously affects patients' postoperative productivity and quality of life [1, 3, 11, 16]. David C. Chen [29] concluded that the occurrence and development of CPIP are independent of technique, but nerve injury is often the result of technical factors, and a deep understanding of the anatomy and nerve identification in the groin area is beneficial in reducing the occurrence of CPIP [2, 18, 24, 55]. Accurate identification allows for correct and rapid intraoperative identification and protection of the nerves in the inguinal region.

**Fig. 9:**
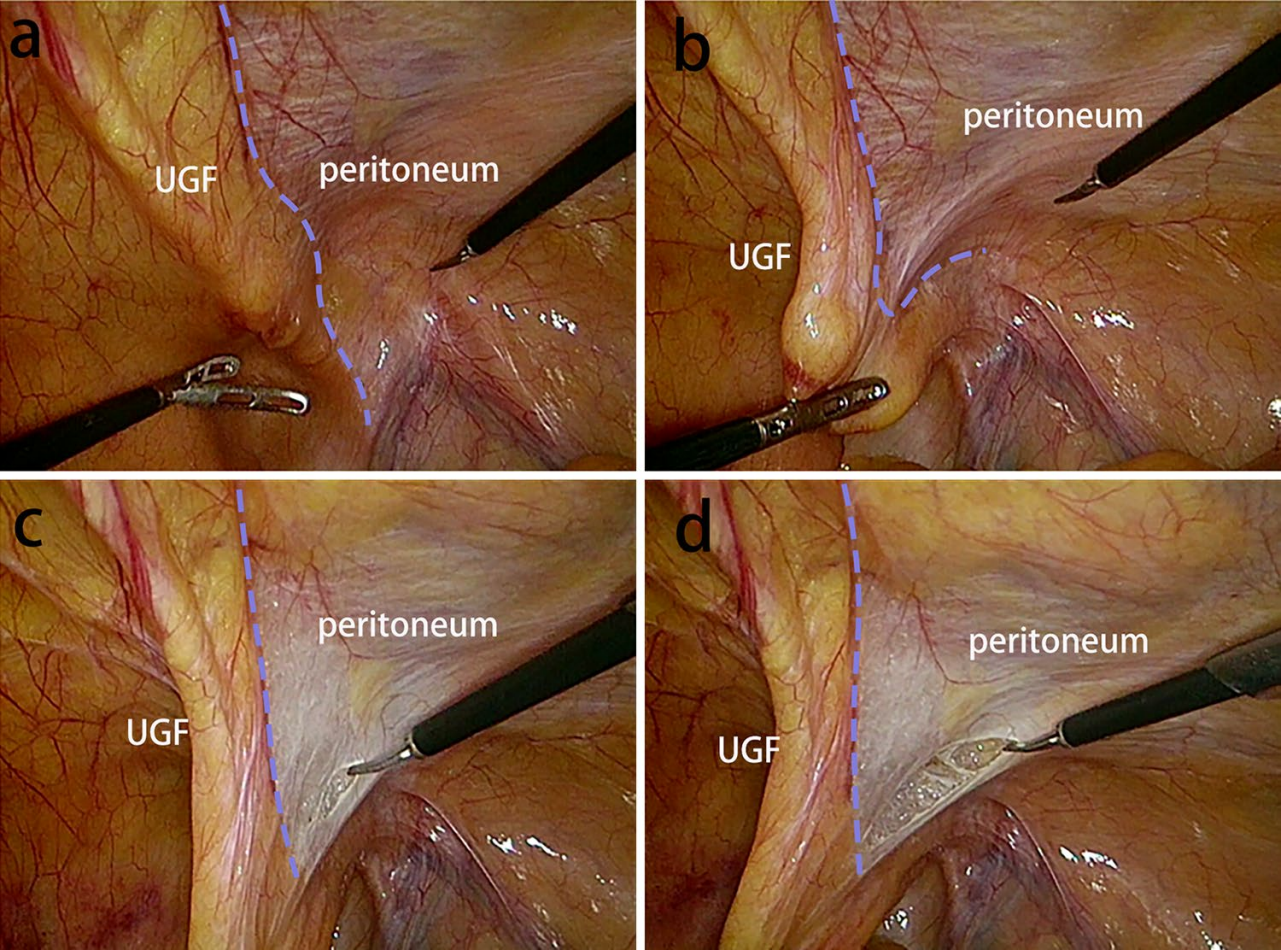
The medial umbilical fold is pulled to reveal the sliding plane between the peritoneum and the UGF. The obvious sliding layer between the extraperitoneal UGF and the surrounding tissues was shown by pulling the medial umbilical fold. The blue dotted line is the boundary of extraperitoneal UGF

**Fig. 10:**
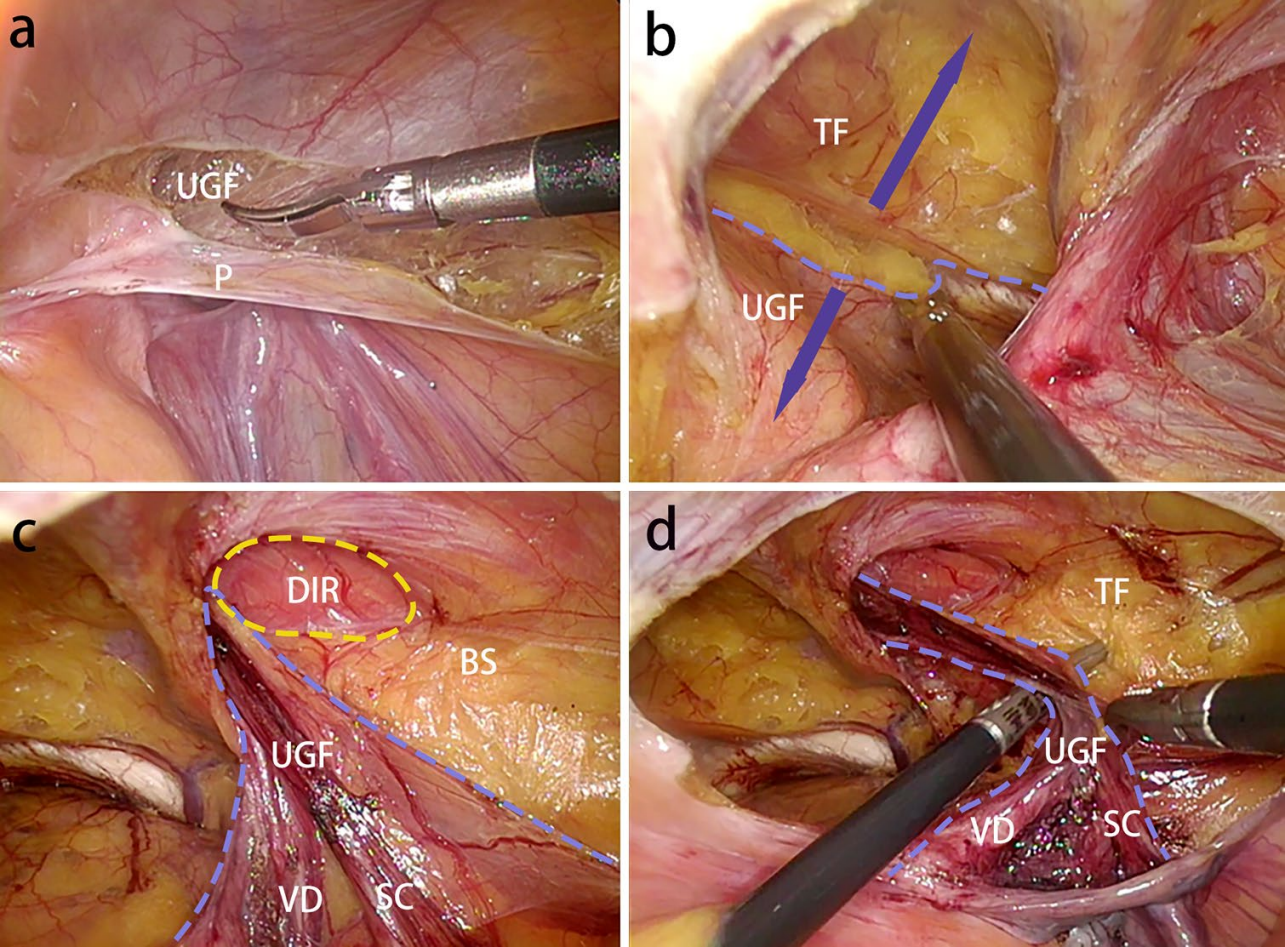
The TAPP was performed through the anatomical understanding of UGF in the preperitoneal space. *UGF* urogenital fascia, *P* peritoneum, *TF* transversalis fascia, *VD* vas deferens, *SC* spermatic cord, *BS* Bogros’ space, *The blue dotted line* the boundary of the extraperitoneal UGF; *yellow circle* deep inguinal ring

**Fig. 11:**
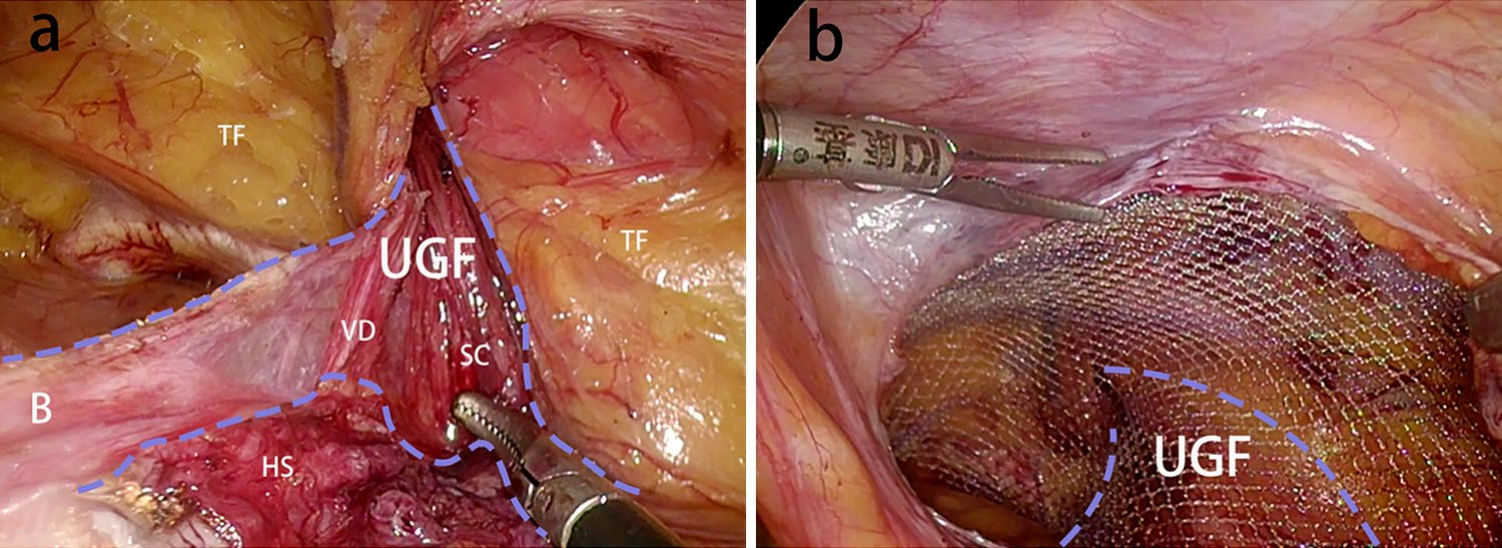
The mesh is placed flat. *UGF* urogenital fascia, *TF* transversalis fascia, *VD* vas deferens, *SC* spermatic cord, *HS* hernia sac, *B* bladder, *The blue dotted line* the boundary of extraperitoneal UGF, *yellow circle* deep inguinal ring

We have summarized the following surgical techniques in laparoscopic transabdominal preperitoneal inguinal hernia repair (TAPP) by understanding the embryonic development and anatomy of the UGF in the preperitoneal space, understanding the surgery, and applying it. Based on the view that the PPF is part of the UGF in the preperitoneal space, the UGF is used instead of the PPF in the following descriptions of surgical procedures. In the TAPP, pulling the medial umbilical fold on one side, it can be seen that the UGF layer and the peritoneal layer appear to slide relative to each other, and repeatedly pulling the medial umbilical fold, one can clearly see the presence of the sliding layer, and at the junction of the sliding of the two layers this first incision of the TAPP is made, so that carbon dioxide gas can fully enter between the two layers, and with the pressure of carbon dioxide gas in the abdominal cavity. The pressure of the intra-abdominal carbon dioxide gas separates the two layers sufficiently to expose the loose connective tissue between the two layers. This helps to find the correct level and to operate faster with less bleeding and damage (Fig. 9). After the incision of the peritoneum, when separating the Bogros’ space, the peritoneum is pulled with one hand and the forceps are operated to carefully separate the peritoneum from the anterior UGF, and the loose connective tissue between the UGF and the peritoneum can be seen (Fig. 10a). When separating the medial Retizus’ space, the UGF is separated within the loose connective tissue between the UGF and the transverse fascia, and the UGF is pivoted posteriorly and the transverse fascia is pushed anteriorly, allowing for rapid blunt sharp union and freeing of the Retizus’ space (Fig. 10b). After the hernia sac is completely freed, the intact UGF anterior to the hernia sac can be seen. In men, the spermatic cord and vas deferens can be seen to be encircled by the UGF and penetrate the deep inguinal ring, while the lateral border of the UGF is also clearly visible when the Bogros’ space is separated laterally (Fig. 10c). The UGF is independent of the transverse fascia and, after being free intact, is lifted without adhesion to the anterior abdominal wall and can be passed from side to side (Fig. 10d). Medially, the UGF continues to wrap around the bladder and forms a flap on the bladder surface. During the TAPP procedure, the displaced portion of the UGF is encountered when separating the medial Retizus’ space, and only by cutting the displaced portion can the hernia repair patch be placed in a spreading position (Fig. 11).

## Conclusion

In summary, we can assume that PPF is continuous with the retroperitoneal renal fascia, ureter and its accessory vessels, lymphatic vessels, peritoneum of the bladder, internal spermatic fascia, and other peritoneal and pelvic urogenital organ surfaces, which means that UGF is a complete fascial system derived from the intermediate mesoderm, which migrates into PPF in the preperitoneal space and the internal spermatic fascia in the inguinal canal.

The study of surgical membrane anatomical processes cannot be separated from the knowledge and understanding of the dynamic process of embryonic development. A holistic view of embryonic development can provide a prospective and demonstrative role in the understanding of the fascial origin, distribution, and alignment. This research method can also guide the innovation of surgical approaches, facilitate the intraoperative search for safe and easily separated surgical planes, improve the speed of surgery while reducing surgical side injuries, and thus promote rapid postoperative recovery. These benefits are consistent with the concept of enhanced recovery after surgery (ERAS).

## Data Availability

Not applicable.

## Abbreviations

LIHR: Laparoscopic inguinal hernia repair
PPF: Preperitoneal fascia
UGF: Urogenital fascia
CPIP: Chronic pain after inguinal hernia repair
ERAS: Enhanced recovery after surgery
TAPP: Transabdominal preperitoneal
TF: Transversalis fascia
VD: Vas deferens
SC: Spermatic cord
BS: Bogros’ space
DIR: Deep inguinal ring
B: Bladder
HS: Hernia sac
P: Peritoneum
UGFV: Urogenital fascia vessels
TFV: Transversalis fascia vessels
